# The FOXP3 rs3761547 Gene Polymorphism in Multiple Sclerosis as a Male-Specific Risk Factor

**DOI:** 10.1007/s12017-018-8512-z

**Published:** 2018-09-18

**Authors:** Natalia Wawrusiewicz-Kurylonek, Monika Chorąży, Renata Posmyk, Olga Zajkowska, Agata Zajkowska, Adam Jacek Krętowski, Joanna Tarasiuk, Jan Kochanowicz, Alina Kułakowska

**Affiliations:** 1 Department of Endocrinology, Diabetology and Internal Medicine, Medical University of Bialystok, Sklodowskiej-Curie 24A, 15-276 Białystok, Poland; 2 Department of Clinical Genetics, Medical University of Bialystok, Białystok, Poland; 3 Department of Neurology, Medical University of Bialystok, Białystok, Poland; 4 Department of Perinatology, Medical University of Bialystok, Białystok, Poland; 5 Faculty of Applied Informatics and Mathematics, Warsaw University of Life Sciences SGGW, Warsaw, Poland; 6 Clinical Research Centre, Medical University of Bialystok, Białystok, Poland

**Keywords:** Multiple sclerosis, FOXP3 gene, Polymorphism, Treg cells, Autoimmune disease

## Abstract

The FOXP3 gene encodes a transcription factor and is predominantly expressed in the CD4^+^CD25^+^ regulatory T cells which plays a pivotal role in the maintenance of immune homeostasis. The defect of FOXP3 gene may provide a critical link between autoimmunity and immune deficiency. The purpose of our study was to evaluate the association of chosen polymorphisms of FOXP3 gene (rs3761549, rs3761548, rs3761547) with different clinical multiple sclerosis (MS) data of our relapsing-remitting groups of patients and in control group. The study was performed on a group consisting of 174 relapsing-remitting MS patients, diagnosed under 40 years of life, and 174 healthy volunteers. Genotyping was performed using a real-time PCR-based method by TaqMan Assays. Significant differences in distribution of allele C rs3761547 were found in male MS patients in comparison to the male healthy group (*p* = 0.046, OR 1.95, CI 95%). No association between MS and the other two polymorphisms was observed in males and females of both studied groups. Our data may suggest that FOXP3 rs3761547 gene polymorphism are related notably with the increased risk of MS development in males patients. To our knowledge this is the first study which indicates gender-specific relation between rs3761547 FOXP3 gene polymorphism and multiple sclerosis.

## Introduction

The FOXP3 gene (ID: 50943), forkhead box protein 3, a member of transcription factor winged-helix family, is located on chromosome Xp11.23 within the area of AIDs (autoimmune diseases) linkage. It is found that FOXP3 gene is mostly expressed in the CD4^+^CD25^+^ regulatory T cells (Treg), and the surface expression of FoxP3 in CD4^+^CD25^−^ T cells is adequate to modify them into Treg with significant inhibition activity (Khattri et al. 2003; Hori et al. 2003; Fontenot et al. 2003). The expression of FOXP3 in CD4^+^ T cells is related to their possibility to function as regulatory T cells (Walker et al. 2003). The many signals that infer the expression of FOXP3 have been described, but the exact mechanisms by which the expression of this protein is controlled in Treg cells are not well understood. It has been described that the synergistic operation of signals downstream of the T-cell receptor (TCR), costimulatory molecules, and cytokine receptors is required for the inclusion of transcription of FOXP3 (Huehn et al. 2009).

Multiple sclerosis (MS), like many other autoimmune diseases, is thought to consequence from a failure of the regular immune tolerance mechanisms, which correspond to pathogenic T cells directed vs self-antigens. T regulatory cells (CD4^+^CD25^+^) play an essential role in the inflection of potentially self-reactive T-cell clones, as they significantly inhibit the immune reply of autoreactive T cells (Maloy and Powrie 2001; Sakaguchi et al. 2001). Notwithstanding, identifying evident failure in Treg-mediated immune control in the widespread human autoimmune disorders has been difficult. This may result from the diversity of the phenomenon of human autoimmunity and from the fact that both genetic and environmental factors are responsible for the disease. Furthermore, a lot of research estimated the number of CD4^+^CD25^+^ and FOXP3^+^ T cells in peripheral blood mononuclear cells of patients diagnosed with MS, T1D, and RA, and found no distinctions in the frequency of these cells towards with reference group (Brusko et al. 2005; Cao et al. 2003; Feger et al. 2007; Haas et al. 2005). However, a number of studies showed alterations in the frequency of FOXP3^+^ T cells in these disorders. In MS, both an magnification (Kumar et al. 2006) and reduction (Venken et al. 2008) in FOXP3^+^ Treg were observed, and in rheumatoid arthritis and newly diagnosed T1D (type 1 diabetes), an increase in FOXP3^+^ Treg was found (Han et al. 2008; Marwaha et al. 2010). The Treg cells with reduced mRNA expression of FOXP3 gene have reduced amount of the Treg celldistinctive genes like CTLA4, EBI3, interleukin 10, and ENTPD1, and increase the expression of T effector cytokine genes such as IFN gamma, TNF alfa, interleukins 4 and 17 (Williams and Rudensky 2007; Wan and Flavell 2008; Zhou et al. 2008; Gavin et al. 2007). Mutations or SNP (single nucleotide polymorphism) of FOXP3 gene may modify its role functionally or quantitatively, therefore leading to the absence of functional CD4^+^CD25^+^ Tregs, resulting in some autoimmune diseases (Wildin et al. 2002), such as immunodysregulation, polyendocrinopathy, enteropathy, X-linked IPEX (immunodysregulation polyendocrinopathy enteropathy) syndrome (Vliet and Nieuwenhuis 2007), type 1 diabetes (T1D) (Bassuny et al. 2003), and autoimmune thyroid diseases (Ban et al. 2007). Polymorphisms have been described in various regions of the FOXP3 gene, such as the promoter, intron, and exon regions. The promoter is highly conserved part of the FOXP3 gene, which is situated 6.5-kb upstream of the first coding exon of FOXP3. This region consists of characteristic TATA and CAAT-box sequences which is activated after NFAT (nuclear factor of activated T lymphocytes) and AP1 (activator protein 1) binding to the TCR receptor (Mantel et al. 2006). Therefore, FOXP3 is an appropriate candidate gene to play a role in organ-specific autoimmune diseases, in particular T1D, thyroid autoimmunity and multiple sclerosis. MS is one of the leading neurodegenerative causes of physical disability. Heterogenetic background of autoimmunity pathway components has been suggested in MS pathogenesis. The purpose of our research was confirm and understand the potential role and relationship of chosen polymorphisms of FOXP3 gene (rs3761549, rs3761548, rs3761547) with different clinical MS data of our relapsing-remitting MS groups of patients and in unrelated group. This is the first study of MS patients in the Eastern part of the Polish population.

## Materials and Methods

The study was conducted as per the revised Helsinki declaration following approval of ethics committee (in Medical University of Bialystok) of hospital from where samples were collected. Informed consent was obtained from patients. Study population consisted of 174 unrelated patients (124 women and 50 men) with clinically defined relapsing—remitting MS according to McDonald’s criteria. All of them were diagnosed under 40 years of life and treated with interferon β (a/b), glatiramer acetate, natalizumab, or fingolimod. The clinical characteristics of all MS patients, men and women separately, are presented in Table 1. 174 Healthy volunteers (mean age 38.7 ± 1.23; 85 woman and 89 men) as a control group with no family history of any autoimmune diseases.

**Table 1:** Clinical data of MS patients

Characteristics	MS patients, *n* = 174	Women, *n* = 124	Men, *n* = 50
Age at onset (years), mean ± SD	41.14 ± 0.79	42.78 ± 0.98	37.14 ± 1.11
Disease duration (years), mean ± SD	8.12 ± 0.42	8.18 ± 0.51	7.96 ± 0.75
EDSS (mean ± SD)	1.85 ± 0.10	1.84 ± 0.12	1.87 ± 0.19

### DNA Isolation and SNP Detection

DNA was extracted from the peripheral whole blood leukocytes using two extraction methods, the salting-out and the membrane method column separation (QIAamp DNA Blood Mini Kit, Qiagen, Germany). Both were performed in agreement to the manufacturer’s protocols. The purity of DNA was determined based on evaluation of the optical density ratio 260/280 nm.

The single nucleotide polymorphism analysis with was performed using the 7900HT Fast Real-Time PCR System (Applied Biosystems, USA). All SNPs in FOXP3 gene (rs3761549, rs3761548, rs3761547) were genotyped by fluorogenic TaqMan SNP technology from ready to use assays library (Applied Biosystems, Foster City, CA, USA) with the TaqMan Genotyping Master Mix (Applied Biosystems, Foster City, CA, USA) in 20 µl reaction volume. The final concentration of genomic DNA for all samples in the experiment sample was 10 ng/µl. The reactions were carried out under the following conditions: 10 min at 95 °C for starting polymerase activity, 40 cycles of 92 °C for 15 s and 60 °C for 1 min. SNPs analysis was performed in duplicate. As a negative control, a sample without DNA was used. The negative control was used to measure any false positive signal caused by contamination.

### Statistical Analysis

Since our sample is small, for all the comparisons in the paper we use Chi^2^ test confirmed with Fisher’s exact test to confirm the results. We report *p*-values for the latter. If possible we report also the odds ratios (OR). *P*-value below 0.005 was considered as statistically significant. The Stata 15 software was used for all the calculations.

## Results

The rs3761549, rs3761548, and rs3761547 polymorphisms of the FOXP3 gene were compared and analyzed between the relapsing—remitting 174 MS patients and 174 healthy individuals. The comparison of clinical parameters between male and female MS patients revealed no notably differences in age at disease onset and EDSS (Expanded Disability Status Scale).

In our study we found that in MS female patients with CC–TT–TT haplotype, of rs3761547, rs3761548, and rs3761549 polymorphisms occurred statistically significantly more frequently in the first generation of neoplastic diseases in the family compared to patients with MS without haplotype (*p* = 0.038). We have also observed that the allele C in rs3761547 was more frequent in male patients with MS in comparison to healthy males (18% vs 10.11%, *p* = 0.046) (Table 2) with OR 1.95 and 95% confidence interval for OR 0.97–3.91. In the group of MS and healthy female we do not observe any result in this polymorphism. There were no differences in other polymorphisms rs3761548 and rs3761549 in FOXP3 gene between males and females in both studied group (Table 2). Some interesting dependences for distribution of the analyzed allele and different variables (sex, type of medication taken, drug change, family disorders) were observed. The presence of a risk allele T of rs3761548 is associated with family appearance (first generation) of MS in male patients with multiple sclerosis (72.22% vs 39.13%, *p* = 0.0065). We have also observed that the same allele T tends to be more frequent in males MS patient who have a vascular diseases in comparison to MS males without it (62.50% vs 39.29%, *p* = 0.0248). Male MS patients with the presence of a combination of C and T alleles of polymorphisms rs3761547 and rs3761549 are statistically more likely to have infectious diseases in comparison to men without these alleles (*p* = 0.035). The MS men who had haplotype C–T–T of polymorphisms rs3761547, rs3761548 and rs3761549 significantly more frequently were treated with glatiramer acetate in comparison to men treated with interferon or natalizumab (*p* = 0.025). All these observations were identified only in the male group of MS patients.

**Table 2:** Distributions of genotypes and alleles of rs3761549, rs3761548, and rs3761547 in multiple sclerosis and healthy groups

SNP	MS group		Control group		*P* (95% CI)
	Female	Male	Female	Male	
rs3761549					
AA					
GA	25 (20.16%)		15 (17.65%)		NS
GG	99 (79.84%)		70 (82.35%)		NS
A		8 (16%)		10 (11.24%)	NS
G		42 (84%)		79 (88.76%)	NS
rs3761548					
GG	44 (35.48%)		26 (30.59%)		NS
GT	57 (45.97%)		34 (40%)		NS
TT	23 (18.55%)		25 (29.41%)		NS
G		31 (62%)		59 (66.29)	NS
T		19 (38%)		30 (33.71%)	NS
rs3761547					
CC					
CT	27 (21.77)		13 (52.29%)		NS
TT	97 (78.23%)		72 (84.71%)		NS
C		9 (18%)		9 (10.11%)	*p* = 0.046*
T		41 (82%)		80 (89.89%)	NS

*OR 1.95 and 95% confidence interval

None of the typically assumed genetic models (codominant, recessive, or dominant) sufficiently fitted the data. This may imply MS has more sophisticated mechanism and SNPs analysis might not be enough to explain it.

The deviation of genotype distribution from Hardy–Weinberg equilibrium was analyzed only in female because of males carry only one copy of the X-chromosome. No remarkably deviation from it was observed for the SNPs.

## Discussion and Conclusions

The etiology of MS is still unclear, but it has been suggested to be influenced by both environmental and genetic factors and its complex interaction. A large number of researches suggest a multifactorial etiology on the basis of genetic susceptibility (Oksenberg et al. 2001). FOXP3 gene is a good candidate gene to play a role in organ-specific autoimmune diseases, due to its expression in Treg cells responsible for the regulation and maintenance of the immunological homeostasis (Fontenot et al. 2003). FOXP3 is a forkhead/winged-helix transcription factor, which is characteristically expressed in regulatory T cells (CD4^+^CD25^+^), and can transform naıve T cells to this regulatory phenotype. Moreover, FOXP3 is a crucial regulator of regulatory T-cell development and function. It interacts with multiple transcription factors like nuclear factor-kappa B, (NF-kB), runt-related transcription factor 1 (RUNX1), retinoic acid receptor-related orphan receptors (RORs) (RORα and RORγT), IFN regulatory factor 4 (IRF4), signal transducer and activator of transcription 3 (STAT3), and Jun (Bettelli et al. 2005; Wu et al. 2006; Ono et al. 2007; Du et al. 2008; Chaudhry et al. 2009).

SNPs in FOXP3 gene in the promoter, introns, and exons regions have been analyzed in the group of autoimmune diseases, like T1D, thyroid autoimmunity, allergic rhinitis (Oda et al. 2013). This polymorphisms may presumably change gene expression by modifying the binding specificity of transcription factors to their binding sites and by altering the kinetics of transcription initiation (Hanel et al. 2011). Moreover, SNPs in FOXP3 gene can influence miRNA, gene splicing or encoded protein structure and activity (Marques et al. 2015). We analyzed three of the known five polymorphisms the promoter region of FOXP3: − 2383C/T (rs3761549), − 3279G/T (rs3761548), and − 3499T/C (rs3761547). Among these three polymorphisms, only the functional effect of rs3761548 on gene expression has been described. The presence in the genotype of the AA homozygote prevents the attachment of certain transcription factors such as E47 and C-Myb, which in effect inhibits FOXP3 transcription. Furthermore, studies by Shen et al. showed that the A allele of this polymorphism is associated with a decrease in luciferase activity against the C allele (Shen et al. 2010). Several studies suggested that this SNP is associated with an increased risk of psoriasis (Gao et al. 2015), Behcet’s disease (Hosseini et al. 2015), vitiligo (Jahan et al. 2013) and development of Graves’ disease (Inoue et al. 2010). A newly meta-analysis of susceptibility of various autoimmune diseases indicated that rs3761548 might be associated with autoimmune (He et al. 2013). The functional effect of other analyzed SNPs is unclear. However, two studies reported connection of rs3761547 with vitiligo (Baranzini et al. 2009) and psoriasis (Gajdošechová et al. 2017).

In our study we investigated whether there is an association of rs3761547 polymorphism in FOXP3 gene with the pre-disposition to multiple sclerosis in the Polish population. We found that the distribution of allele C of this polymorphism differed notably between male relapsing-remitting MS patients and the male control group. This result may suggest that rs3761547 polymorphism gene could contribute to MS development in males. This observation is especially interesting and even unique because men suffer from autoimmune diseases less frequently. The prevalence of women with autoimmune diseases is probably related to the immunomodulatory role of estrogens. Moreover, the male are a hemizygote. Woman have two copies of chromosome X but only one of them is active and the second one is inactivated during the lyonization process. Additionally, we have observed that none of the two other polymorphisms rs3761548 or rs3761549 contributes to the genetic predisposition to MS in the Polish population. Furthermore, the analysis of clinical data of the patients with MS showed the existence of some of their relationships with the studied polymorphisms. The presence of a risk allele T of rs3761548 polymorphism was associated with family appearance of MS in male patients with multiple sclerosis. Moreover, the MS men who had a combination of alleles C–T–T of polymorphisms rs3761547, rs3761548, and rs3761549 remarkably more frequently were treated with glatiramer acetate in comparison to men treated with interferon or natalizumab. To our knowledge, this is the first study to investigate the relationship between FOXP3 polymorphisms and the risk of the development of MS in Poland. Some of our results are consistent with the previously described studies. Branzini et al. reanalyzed the data from 16 genome-wide association studies (GWAS) of different autoimmune diseases and found no evidence of association of FOXP3 gene polymorphisms with MS in Caucasian populations of European origin (Baranzini et al. 2009). Similar results were obtained by Gajdošechová et al. in the Slovak population (Gajdošechová et al. 2017). However, other authors found a connection between FOXP3 rs3761548 and multiple sclerosis in population of Iran (Eftekharian et al. 2016; Jafarzadeh et al. 2015). The relationship between polymorphism rs3761547 has been demonstrated only in the case of other autoimmune diseases, but not in multiple sclerosis (Bassuny et al. 2003; Ban et al. 2007; Baranzini et al. 2009; Gajdošechová et al. 2017). Similarly, in the case of rs3761549 polymorphism Bossowski et al. have demonstrated the relationship between this polymorphism in female with Grave’s disease in the Polish population (Bossowski et al. 2014). Discrepancies between the published data and our results may result from many aspects. The most notably are inter-ethnic and geographical genetic differences. This may depend on the allele frequency, genome location on chromosomes, various LD patterns (linkage disequilibrium) in the populations, various evolutionary histories of genes affecting complex diseases, and influence of various environmental factors. Differences in the results may also be related to the small number of the studied groups, which leads to the lack of statistical significance of the effects. In addition, the research design and criteria for the inclusion of patients may disturb the overall statistical approach. However, replication in a larger sample set and other populations are required in order to confirm these findings and it is still required to increase our knowledge-base for this gene.

In conclusion, our data suggest that FOXP3 rs3761547 gene polymorphism is related notably to the increased risk of relapsing-remitting MS development in males patients. To our knowledge this is the first study which indicates gender-specific relation between rs3761547 FOXP3 gene polymorphism and multiple sclerosis. This investigation could further enhance a better understanding of the role of the molecular factors in pathogenesis of MS. Moreover, it can help to develop more efficient clinical methods to control immune responses through the targeting Treg suppressive functions. FOXP3 is an aspirant-gene for understanding the underlying pathogenesis of autoimmune diseases, mainly through its known role in the regulation of immune processes and the fact that that mutations of this gene are associated with the development of X-linked immunodysregulation polyendocrinopathy enteropathy syndrome.
